# Collective negative shocks and preferences for redistribution: Evidence from the COVID-19 crisis in Germany

**DOI:** 10.1007/s10888-022-09558-2

**Published:** 2023-03-18

**Authors:** Bellani Luna, Fazio Andrea, Scervini Francesco

**Affiliations:** 1 Cluster of Excellence “The Politics of Inequality” and Department of Economics, University of Konstanz, Konstanz, Germany; 2 IZA, Bonn, Germany; 3 AXA Research Lab on Gender Equality of Dondena Research Center, Bocconi University, Milan, Italy; 4 Department of Political and Social Sciences, University of Pavia, Pavia, Italy

**Keywords:** Preference for redistribution, Inequality aversion, COVID-19, D31, D63, D72

## Abstract

Using new data from a three-wave panel survey administered in Germany between May 2020 and May 2021, this paper studies the impact of a negative shock affecting all strata of the population, such as the development of COVID-19, on preferences for redistribution. Exploiting the plausibly exogenous change in the severity of the infection rate at the county level, we show that, contrary to some theoretical expectations, the worse the crisis, the less our respondents expressed support for redistribution. We provide further evidence that this is not driven by a decrease in inequality aversion but might be driven by the individuals’ level of trust.

**JEL Codes** D31 · D63 · D72

We thank the editor and three anonymous referees for insightful comments and suggestions that greatly improved the paper. We are grateful to Thomas Wöhler for coordinating the data collection. We would like to thank Martin Wessel and Moritz Seebacher for their excellent research assistance. This project is funded by the Deutsche Forschungsgemeinschaft (DFG - German Research Foundation) under Germany's Excellence Strategy-EXC-2035/1 – 390681379, as well as a financial contribution from the Italian Ministry of University and Research PRIN 2017K8ANN4 "New approaches to political economy: from methods to data" is gratefully acknowledged. Usual disclaimers apply.

✉ Scervini Francesco
francesco.scervini@unipv.it

Bellani Luna
luna.bellani@uni.kn

Fazio Andrea
andrea.fazio@unipv.it

## 1 Introduction

Income and wealth distribution varies substantially between countries (Piketty 2018); part of this heterogeneity can be explained by a difference in preferences for redistribution because individuals' support for income redistribution crucially influences the implementation of redistributive policies (Alesina and Giuliano 2011; Alesina et al. 2004; Alesina et al. 2001). However, understanding what affects people's demand for income redistribution remains a challenge. Preferences for redistribution depend on several factors such as self-interest (Meltzer and Richard 1981), fairness concerns (Alesina and Angeletos 2005; Benabou and Tirole 2006), trust in institutions (Algan et al. 2011; Kuziemko et al. 2015), and macroeconomic factors (Giuliano and Spilimbergo 2014; Roth and Wohlfart 2018), to name a few.

To explore further how these preferences are formed and, possibly, changed, we study the impact of a negative shock affecting all strata of the population, such as the development of COVID-19, on preferences for redistribution.

Previous research suggests that such unexpected events affect people's attitudes towards redistribution through different drivers. One possible channel through which unexpected shocks influence preferences for redistribution is by shifting people's normative views. Research shows that fairness concerns and beliefs about luck and merit affect preferences for redistribution (Alesina and Angeletos 2005; Benabou and Tirole 2006; Piketty 1995). If people believe that income inequality is the outcome of a fair, meritocratic process, they are less likely to support redistribution. In contrast, if people believe that inequality is due to causes beyond individuals' control, they demand more redistribution (Almås et al. 2010, 2020; Cappelen et al. 2007). Gualtieri et al. (2019) use Italian data to test this channel and show that the experience of an earthquake increases support for redistribution even among those who do not incur any material damage. This result is explained by the fact that an exogenous negative shock might push people to believe that economic success is mainly due to random luck rather than merit, thus increasing demand for redistribution. Another driver of redistributive preferences is insurance against labor or income shocks. Supporting this line of thought, Esarey et al. (2012) show in a laboratory experiment that people demand more redistribution when facing a probable (negative) income shock. However, when the probability of the random shock is moderate, only left-oriented individuals support income redistribution. Finally, the literature on preferences for redistribution shows that people support income redistribution if they have sufficient trust in the government (Kuziemko et al. 2015). Handling unexpected crises poses a challenge to political institutions, and people might mistrust institutions if they feel that crises of this type are mismanaged. In this regard, Daniele et al. (2020) show the effects of the current pandemic crisis on political opinions with a survey experiment. The authors find that the COVID-19 crisis decreases the willingness to support the welfare state, and it also decreases both trust in institutions and interpersonal trust. Overall, the literature shows that an unexpected negative shock such as a natural disaster or a pandemic might have contrasting effects on preferences for redistribution. If a natural disaster pushes people to think that economic success ultimately depends on luck rather than effort, we might expect an increase in support for redistribution. However, if a natural disaster induces people to lose trust in the government, the demand for redistribution might decrease.

To show how the pandemic shock might affect support for redistribution, we run a survey on a sample of around 3,000 individuals in Germany at three different stages of the crisis. The timing of our survey allows us to take advantage of the unexpected increase in the number of deaths and cases that have hit Germany since autumn 2020. The first wave of our survey was collected in late May 2020, when Germany reported a relatively low number of deaths and infections due to the new virus. The second wave was collected at the beginning of November 2020, when COVID-19 started to hit Germany hard. Indeed, the number of deaths related to the virus doubled in November compared with May, and starting from December 2020, Germany was forced to implement a strict lockdown. Finally, the third wave was collected in May 2021, when COVID-19-related cases and deaths were decreasing but were still high compared with the previous year.

To identify the effect of the pandemic on individual preferences, we link data on the number of COVID-19 cases at the county (Landkreis) level with individual data on preferences for redistribution. We include in our regressions a set of variables to control for subjective assessments about the COVID-19 crisis, and we include some standard socio-demographic controls, such as employment conditions, that might be affected by the crisis. Furthermore, and most importantly, the panel structure of our data allows us to control for individual unobservable heterogeneity. We find that an increase in the number of COVID-related cases leads to a decrease in support for redistribution. Thanks to the specificity of our survey, we are also able to investigate several aspects that might drive our results. We suggest that the decrease in support for redistribution is not driven by people becoming less inequality averse, as we also find that an increase in the number of COVID-related cases leads to an increase in inequality aversion. Rather, we find that an increase in the number of COVID-related cases leads to a decrease in trust toward the institutions. This might indicate that at least part of the lower support for redistribution is driven by a general decrease in institutional trust. We further investigate the role of trust in determining preferences for redistribution, and we find that our results are driven by individuals with a low level of trust. In contrast, among people with a high level of trust, an increase in COVID-19 cases has either no or even positive effects on support for redistribution. Overall, our results suggest that the pandemic crisis might have affected both people's redistributive preferences and normative views about inequality in an opposite way.

Our findings contribute to three strands of the literature. First, we add to the literature studying individual preferences for redistribution. The standard model by Meltzer and Richard (1981) suggests that people below the median income demand more redistribution because they can benefit from it. However, self-interest alone cannot provide a complete explanation of the functioning of redistributive preferences as they are influenced by several factors (Alesina and Giuliano 2011; Corneo and Grüner 2002; Fong 2001; Sabatini et al. 2020; Scervini 2012). We add to this complex puzzle by pinpointing the different effects that a disaster with roots in both natural causes and government response, such as a pandemic crisis, might have on support for redistribution, trust in institutions and inequality aversion. Second, we contribute to the literature linking the COVID-19 crisis to individual preferences and political attitudes. Research has shown that the pandemic crisis affects people's views on a number of outcomes such as trust in national governments (Esaiasson et al. 2020; Fazio et al. 2022; Hensel et al. 2022; Lazarus et al. 2020; Saka et al. 2022), economic anxiety (Fetzer et al. 2020) and support for safety-net programs (Balasundharam et al. 2021; Rees-Jones et al. 2020). Asaria et al. (2021) investigate the effect of the pandemic shock on income and health inequality aversion in the United Kingdom, Italy, and Germany. The authors find that reporting a health or income shock due to the pandemic is associated with lower inequality aversion. However, when implementing a difference-in-differences analysis, the exposure to health or income shocks affects inequality aversion only for individuals belonging to high-risk groups. Cappelen et al. (2021) show through a survey experiment in the United States that the COVID-19 crisis makes people more willing to accept inequalities due to luck.

Because the moral views on inequality are usually related to preferences for redistribution, the authors suggest that this shift in moral views might affect support for redistributive policies. Our main contribution is to provide a broad and consistent picture of the way in which the pandemic might affect redistributive preferences and inequality aversion. Last, we contribute to the literature on natural disasters and individual attitudes. Natural disasters affect individual attitudes such as trust and risk attitudes (Bourdeau-Brien and Kryzanowski 2020; Cassar et al. 2017; Hanaoka et al. 2018) and voting behavior (Goebel et al. 2015; Masiero and Santarossa 2020). We add to this literature by showing the effects of a pandemic crisis on individual redistributive preferences. The paper develops as follows: the next section briefly summarizes the COVID-19 pandemic in Germany. Section 3 describes the data and the main variables we use. Section 4 depicts our identification strategy. Section 5 presents the results and some robustness tests, while Section 6 concludes the paper.

## 2 COVID-19 in Germany: January 2020–May 2021

Our empirical strategy builds on the marked differences in the level of diffusion of the COVID-19 virus in Germany between March 2020 and May 2021. In this section, we briefly recall the development of the pandemic crisis in Germany during this time span. Figure 1 presents the daily trends of the two main indicators: the number of new cases and the number of deaths.

### 2.1 First Wave

The first case of COVID-19 infection was reported on 27 January 2020 in Bavaria. However, the infection did not spread significantly until March 2020. The first death due to COVID-19 was reported on 8 March, and on 10 March, Coronavirus infections were reported in all 16 German federal states. As COVID-19-related cases and deaths rose, the authorities started to implement restriction measures such as travel bans, school, restaurant, and cinema closures and quarantine measures. To sustain the economy, the federal government launched a relief program worth 156 billion euros. During this first wave, the number of cases reached a peak at an infection rate of 44.7 reported infections per 100,000 inhabitants on 4 April 2020. The infection rate then fell again until it dropped to under 10 on 3 May. The death rates in the states rose and fell relatively similar to the infection rates and reached a peak in the first wave on 18 April with a national death rate of 236 (on a 7-day average). Overall, the first wave of COVID-19 in Germany was relatively mild compared with other countries such as Italy, France, and England. Indeed, Germany reported a low number of cases and deaths (Stang et al. 2020).

### 2.2 Second wave

From May to mid-August, the daily state infection rates were relatively low and constantly below 10 infections per 100,000 inhabitants, whereas between mid-August and the beginning of October, the infection rates started increasing but stayed below 20 daily infections per 100,000 inhabitants. In October, there was a sharp increase in infection rates all over Germany. Infection rates rose from 17 to 154 daily infections per 100,000 inhabitants in a month. The second wave reached its peak in December with a daily infection rate of over 200, the highest daily infection rate in Germany until November 2021. Infection rates dropped until the middle of February to a daily infection rate of 60.9. COVID-19-related deaths rose sharply with the second wave. The national death rate peaked at 889 daily deaths on a 7-day average. The death rate then sharply fell until April. To halt the spread of the virus, the authorities were forced to implement a "light lockdown" on 2 November 2020. However, the restrictive measures had to be reinforced at the end of November because the cases kept growing. Unlike what happened in the first wave, Germany was hit strongly by the second wave of the pandemic, and the spread of the virus varied substantially from region to region within Germany (Schuppert et al. 2021).

### 2.3 Third wave

Between February and April 2021, infection rates started rising again and peaked on 22 April 2021 with 177 daily infections per 100,000 inhabitants. Infection rates then dropped to around 70 infections per 100,000 at the end of May. Partly also due to vaccinations, the death rate remained relatively low and held at around 200 deaths per day (7-day average). On 23 April 2021, a new federal epidemic law went into place. This new law imposed social contact restrictions and a curfew from 10 pm to 5 am in every district where the 7-day-incidence was above 100 for three days. The new law also allowed for relaxations in districts where the infection rate stayed stable at under 100.

## 3 Data description

Our analysis builds on original survey data collected from a sample of the adult German population at three different stages of the pandemic crisis.[1] Quota sampling has been used to achieve the representativeness of the sample in terms of age, gender, education levels and federal state, with double selection probability for East Germans.[2] The survey was conducted online and administered by the survey firm Kantar. As shown in Fig. 1, the first wave of our survey was collected in the second half of May 2020, the second wave was collected at the beginning of November 2020, and the third wave was collected in the first part of May 2021. Hence, our survey allows us to have data on people's redistributive preferences soon after the end of the first wave, at the beginning of the second wave and at the end of the third wave. The original sample (interviewed in the first wave) is composed of 3,258 individuals. Of those, 2,482 participated in the second wave, 2,248 participated in the third wave, and 2,029 participated in all three waves. After cleaning the data for missing data in our variables of interest or important controls, the number of individuals in our final sample is 1,644 for the balanced panel and 2,266 for the unbalanced panel (Table 1).

[1] For more information on the survey, "Living in Exceptional Circumstances," see Appendix A.
[2] All the descriptive statistics are reported in Table 1.

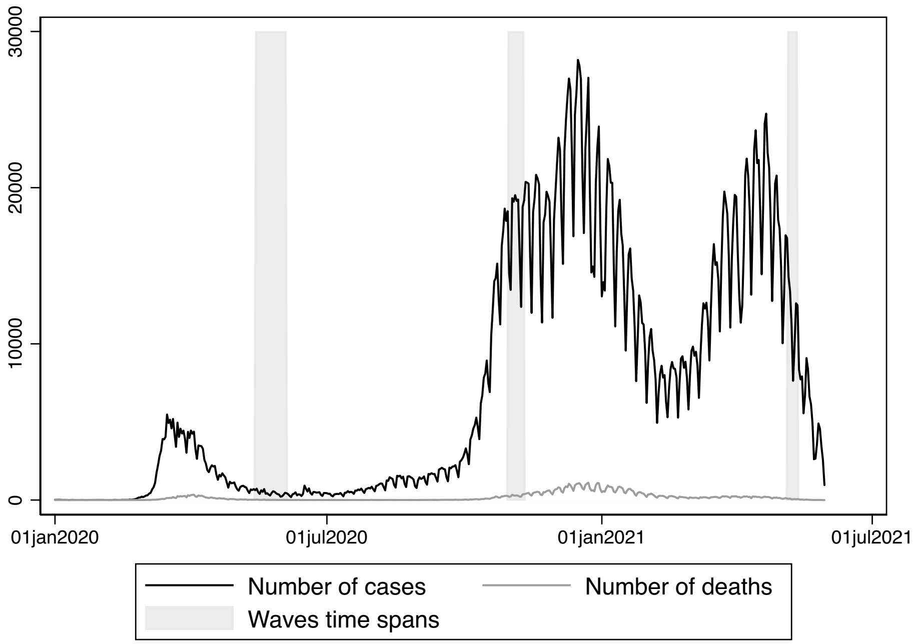

**Fig. 1** COVID-19 and interviews dates

### 3.1 COVID-19 data

To understand the effect of the severity of the pandemic on preferences for redistribution, we match our survey data with data on the number of COVID-19 cases in Germany provided by the Robert Koch Institute (RKI). To measure the incidence of the pandemic, we use the log of the cases and deaths per 100,000 inhabitants. We match individual data with county- (Landkreis) level data on the average number of cases and deaths during the last 7, 14, 28, and 90 days prior to the day of the interview. To give a better idea of the temporal variation in the severity of the crisis that we are using in the paper, Fig. 2 shows the average number of new cases per day during the field period of the three survey waves. We can see that, while almost all counties had an increase in the severity of the health crisis between our first and second wave, a more heterogeneous picture comes from comparing the second to the third wave.

### 3.2 Preferences for redistribution

We build our main measure of preferences for redistribution using answers to the following statement: "It is the role of the state to reduce the income gap between high-income people and those on low incomes." Individuals might agree or disagree with this statement on a 1-7 scale, where 1 corresponds to "totally disagree" and 7 to "totally agree." This measure

**Table 1** Summary statistics

| Variable | Mean | Std. Dev. | Min. | Max. | N |
|---|---|---|---|---|---|
| *Demographics* | | | | | |
| Male | 0.495 | 0.5 | 0 | 1 | 6176 |
| Age | 50.933 | 15.75 | 18 | 92 | 6176 |
| Married | 0.514 | 0.5 | 0 | 1 | 6176 |
| Have children | 0.621 | 0.485 | 0 | 1 | 6176 |
| Tertiary education | 0.343 | 0.475 | 0 | 1 | 6176 |
| Very good or good health | 0.527 | 0.499 | 0 | 1 | 6176 |
| *Employment situation* | | | | | |
| Changed (worsen) | 0.147 | 0.354 | 0 | 1 | 6176 |
| *Covid-19* | | | | | |
| Belong to Corona risk group | 0.366 | 0.482 | 0 | 1 | 6176 |
| Know someone in risk group | 0.829 | 0.376 | 0 | 1 | 6176 |
| Deaths/100,000 inhabitants (log) (last 14 days) | 2.158 | 0.589 | 0 | 2.691 | 6176 |
| Infected / 100,000 inhabitants (log) (last 14 days) | 4.837 | 1.959 | 0 | 7.339 | 6176 |
| *Political and Social Preferences* | | | | | |
| Redistribution (main variable) | 4.263 | 1.802 | 1 | 7 | 6176 |
| Redistribution (alternative variable) | 4.184 | 2.714 | 0 | 10 | 6176 |
| Pie rich | 3.497 | 2.218 | 0 | 10 | 6176 |
| Pie poor | 5.035 | 2.598 | 0 | 10 | 6176 |
| Society is fair if all people have same living conditions | 4.596 | 1.728 | 1 | 7 | 4058 |
| In Germany, people have same opp. in life | 5.32 | 1.265 | 3 | 7 | 5633 |
| In Germany, equivalent life chances for future generations | 5.189 | 1.314 | 3 | 7 | 5633 |
| Trust fed. Gov. | 4.077 | 1.807 | 1 | 7 | 6176 |
| Trust Parliament | 3.915 | 1.7 | 1 | 7 | 6176 |
| Trust state gov. | 4.105 | 1.755 | 1 | 7 | 6176 |
| Interpersonal trust | 4.18 | 2.53 | 0 | 10 | 6176 |

For comparison, the official statistics report the share of males at 49.35%, the average age as 44.5 (including people under the age of 18), while the share of people with tertiary education is 33.5%

is very similar to those used in the literature. For example, Giuliano and Spilimbergo (2014) use answers to the following question: "Some people think that the government in Washington should do everything to improve the standard of living of all poor Americans. Other people think it is not the government's responsibility and that each person should take care of himself. Where are you placing yourself on this scale?"

Analogous questions have been used in other studies to measure preferences for redistribution (Alesina et al. 2018; Guiso et al. 2006; Roth and Wohlfart 2018) and are part of the core module in international surveys such as the European Social Survey. On average, around 34% of our respondents disagree to some extent, 20% neither agree nor disagree, and around 46% agree with the statement.

As a robustness check, we also use answers to the following question: "How much redistribution of income do you support between citizens in Germany? No redistribution means that the state does not interfere in the distribution of income. Full redistribution means that everyone earns the same income after the redistribution has been carried out." Individuals can answer this question on a 0-10 scale where 0 corresponds to "no redistribution"

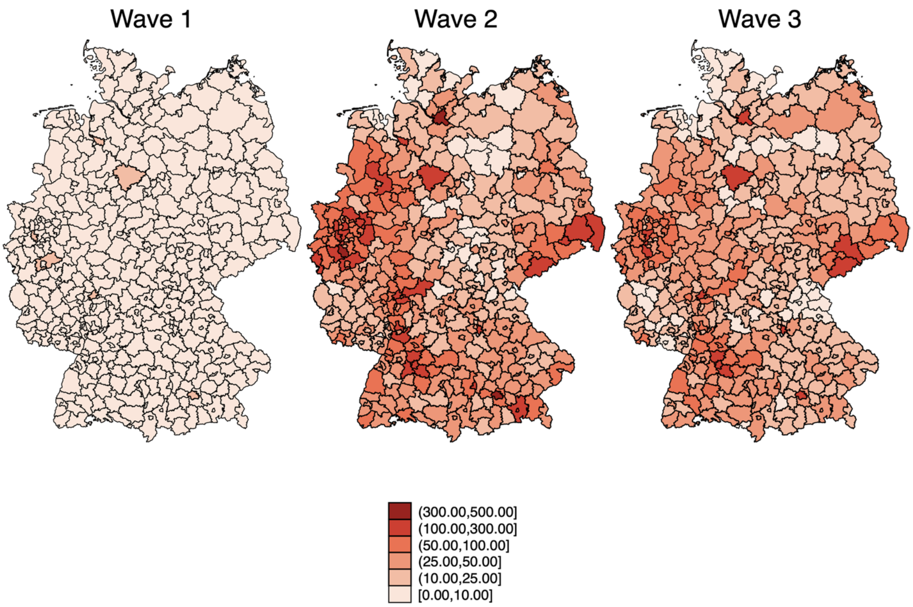

**Fig. 2** Average number of cases per 100,000 inhabitants over each survey field period and 10 to "full redistribution." This latter question focuses more on solidarity among German citizens rather than underlining the role of the state. This slight difference between the questions allows us to better understand possible mechanisms that might explain how the COVID-19 crisis affects redistributive preferences.[3] Questions of this kind are also quite standard in the literature.

### 3.3 Other Variables

Among additional variables that might influence people's redistributive preferences and attitudes, we first consider possible changes in the employment situation. The survey asks individuals whether their employment situation changed due to the pandemic. Possible changes are a decrease/increase in working hours, a worsening of the business condition (only asked of self-employed individuals), the beginning of paid or unpaid compulsory leave, and the occurrence of income losses due to the pandemic (only asked of employed individuals). From this information, we build a dummy variable equal to 1 if the individual has incurred any possible change that might result in a loss of income and 0 otherwise. Around 15% of our sample experienced an income or job loss due to the pandemic.

We also consider the possible impact of the pandemic on preferences for redistribution through individuals' health or the health of friends or family members. To this goal, we include a set of dummy variables to capture the subjective health status and a variable that indicates whether the respondent is considered at risk of severe COVID-19 effects due

[3] The correlation between this variable and our main proxy for preference for redistribution is only 0.5, which suggests that they might capture different aspects of redistribution.

to pre-existing conditions or whether they know someone who is. More than half of our respondents report good or very good health, around 38% of the sample declared belonging to the corona risk group, and only 16% declared not knowing anyone belonging to that group.

Finally, to explore some possible mechanisms behind the impact of the pandemic on preference for redistribution, we have also collected data on *inequality aversion*, *perception of inequality of opportunity*, *interpersonal trust* and *trust in institutions*. To measure inequality aversion, we use the answers to the following statement: "Consider the total income, after taxes, earned by all persons in Germany as a cake with 10 pieces. How many pieces of cake SHOULD ideally be distributed to the 20% of the population who earn the most and the 20% of the population who earn the least?" Answers are on a 0-10 scale where 0 corresponds to "nothing," and 10 corresponds to "the whole cake." The median value for the richest 20% is 4 (standard deviation 2.2), while it is 5 for the poorest 20% (standard deviation 2.6). In the first wave, we also asked the respondents how many pieces of cake they thought those two groups were currently enjoying (the median responses are 7 and 2, respectively). These numbers clearly suggest that our respondents—on average—tend to overestimate both shares and have difficulties taking into account that there is another 60% of the population between the rich and the poor. However, the identifying variation in our preferred specification comes from changes within individuals, so possible cognitive differences across individuals in answering these questions are taken care of. Moreover, using the data from the first wave, we can also see that on average our respondents perceive the society as more unequal than they would want it to be, thinking on average that the richest 20% should get 34% less of what respondents think the richest people are currently receiving, while the poorest 20% should receive 125% more, giving us confidence that within individuals, those two variables are a good proxy for individual normative views on inequality.

We also show results using individuals' agreement, on a 1–7 Likert scale, with the statement that a society can be considered fair if all individuals have the same living conditions. This question was only asked in the first and in the second wave of our survey; therefore, we chose the previous one as our main proxy for inequality aversion.

We use answers to the following two statements to measure the perception of inequality of opportunities: i) In Germany, people have the same opportunities in life to get ahead and ii) In Germany, further generations will have equivalent life chances. These two questions are asked on a 0-10 Likert scale in all the waves. The first question proxies the perception of current inequality in opportunities; the second one gives us a measure of intergenerational inequality in opportunities.

We measure interpersonal trust through answers to the following statement: "Would you generally say that you can trust most people or that you can't be careful enough when dealing with people?" Answers to this question are on a 0-10 scale, with 0 corresponding to "You can't be careful enough" and 10 corresponding to "You can trust most people." This question, developed by Rosenberg (1956), is commonly used to measure trust (Algan et al. 2016; Borghi et al. 2020; Daniele and Geys 2015; Thöni et al. 2012) and is part of the core modules of international surveys such as the World Values Survey and the European Social Survey. The level of trust in our sample is surprisingly low. On the 11-point scale, the average level of interpersonal trust is around 4. Last, we measure trust in institutions through answers to the following statement: "A number of public institutions and institutions are mentioned here. Please tell us how much trust you have in the respective institution in the current crisis situation." Answers are on a 1-7 scale where 1 corresponds to "no trust at all" and 7 to "very high level of trust." We focus in particular on those institutions that are normally responsible for the implementation of redistributive policies, that is, federal and state governments and Parliament. The majority of the respondents trust the federal government, with more than 30% reporting a high or very high level of trust. A similar picture is true for state governments and Parliament, with an average higher trust in the former.[4]

## 4 Identification strategy

Our aim is to identify the causal effect (if any) of a collective shock, such as the COVID-19 pandemic, on preferences for redistribution. To uncover the causal relationship, we exploit the heterogeneous diffusion of COVID-19 over time and across regions that is plausibly exogenous to individual behavior.[5] The main estimation is the following ordinary least squares model (OLS) with individual fixed effect:

$$Y_{i,t} = \alpha + \beta Cases_{i,c,t} + \lambda X_{i,t} + \gamma_i + \varepsilon_{i,t} \tag{1}$$

where $Y_{i,t}$ is the dependent variable, that is the preferred level of redistribution[6] for individual $i$ at time $t$, $Cases_{i,c,t}$ is the logarithm of the average number of COVID-19-related cases per 100,000 inhabitants in the 14 days prior to the interview for the individual $i$ in county $c$, $X_{i,t}$ is a set of variables controlling for potentially confounding factors such as changes in the employment situation due to the pandemic crisis, marital status, health status and possibly belonging to health risk groups. Finally, $\gamma_i$ is the individual fixed effect, and $\varepsilon_{i,t}$ is the idiosyncratic error term. Estimated standard errors are robust to heteroskedasticity. The random and unexpected increase of COVID-19 cases helps us to identify the causal nexus between the COVID-19 pandemic and preferences for redistribution. Furthermore, the inclusion of the individual fixed effect crucially helps us to eliminate all the time-invariant characteristics that could have biased our results, such as personality traits or individual ability. With respect to the meaning of the variable *Cases*, it is worth noting that its causal effect refers not only to the number of cases *per se* but also to all those aspects that are related to the number of cases and difficult to measure and/or highly correlated, such as lockdown measures, psychological anxiety and insecurity, social distancing, recovery measures and so on. Therefore, the interpretation of the coefficient should be broader than the mere effect of an increase of a 1% of COVID-19-related cases.[7] Overall, the variable is a broad proxy for the pandemic-related issues, which is most intuitively measured by the number of newly infected people during the period under consideration.

[4] The correlations between those variables is very high, ranging between 0.8 and 0.9.

[5] With respect to endogeneity, some concerns may arise for omitted variable bias if some unobserved factors might jointly determine propensity to redistribution and the diffusion of the pandemic. However, we rule out most of such issues as follows. First, we include county and individual fixed effects, controlling for all individual and territorial features that are invariant in the very short period under investigation. Second, we use contagion rates at the county level, ruling out any effect of specific types of households (low income, low education, and so on) that may also determine propensity for redistribution. Third, we employ the *variation* and not the *level* of COVID-19 cases. Finally, we also tested for the presence of error correlation within households in the same county (see Table 11), and the results are unaffected.

[6] See the previous section for the description of the dependent variable.

[7] For this reason, we do not include time fixed effects in our regression models, because they would isolate the effects of COVID-19 diffusion per se, disregarding other features of interest for our analysis of newly infected people during the period under consideration.

## 5 Results

In this section, we present and discuss our main results. Table 2 shows the effect of the COVID-19 infection on preference for redistribution. We present different specifications for our results. There is a simple correlation in the first column. We then add individual controls (column 2) and individual fixed effects using both unbalanced (column 3) and balanced (column 4) panels. We find a negative and significant effect of the COVID-19 infection rates on preferences for redistribution.

More precisely, to give a sense of the magnitude of the impact, in our preferred specification, a 1% increase in COVID-19 cases decreases support for redistribution by about 2 percentage points; that is, 0.5% of the sample mean and 1.1% of the standard deviation. The difference in redistributive preference we find in our sample between the low and the highly educated individuals is around 23 percentage points on average, while the average difference in redistributive preferences for individuals aligning with the left and right ends of the political scale is 72 percentage points. Our effect is around 9% and 2.7% of those effects, respectively. If we consider that, given the exponential growth and diffusion of the virus, the real average experienced growth in the severity of the pandemic is much larger than a 1% increase, in fact, as high on average as an 85% increase, the total effect of the pandemic on preferences for redistribution is closer to the difference in redistributive preferences we see among political extremes.

At first glance, this strong negative impact could be seen as puzzling. Theoretically, one could have expected that a shock that affects individuals without distinction based on their effort or deservingness would increase the support for redistribution, both from a societal point of view because it can be seen as fair compensation for undeserved differences and from an individual perspective because redistributive policies can be seen as an insurance mechanism given the higher likelihood of being exposed to a loss oneself. Our findings instead seem to suggest that other mechanisms could be in place, such as an increase in fear and worries, which might bring about more individualism, or a decrease in trust in the system that has not yet managed to put an end to the pandemic, which might reduce the willingness to support any policy that the same system is in charge of.

Most likely, all these mechanisms are at play, and the result we see is a combination of them. To shed more light on the possible channels behind this negative impact of the

**Table 2** Preference for redistribution

| | (1) OLS | (2) OLS | (3) FE unbalanced | (4) FE balanced |
|---|---|---|---|---|
| Incidence rate (last 14 days) | −0.029*** (0.011) | −0.023** (0.011) | −0.018* (0.009) | −0.022** (0.010) |
| Individual controls | No | Yes | Yes | Yes |
| Observations | 7137 | 7035 | 6176 | 4932 |
| Individuals | | | 2266 | 1644 |
| R2 | 0.001 | 0.056 | 0.006 | 0.008 |

The table shows the results of linear regression with and without individual fixed effects. The dependent variable measures preferences for redistribution. The variable of interest is the logarithm of the average number of COVID-19-related cases per 100,000 inhabitants at the county level in the 14 days prior to the interview. Individual controls are gender, age and education groups, partnership/marital status, parental status, health, employment status, and Covid perception

Standard errors in parentheses *** $p < 0.01$, ** $p < 0.05$, * $p < 0.1$

**Table 3** Income losses

| | (1) | (2) | (3) | (4) |
|---|---|---|---|---|
| | FE unbalanced | | FE balanced | |
| | Economic losses | Redistribution | Economic losses | Redistribution |
| Incidence rate (last 14 days) | 0.001** | −0.031** | 0.001 | −0.036** |
| | (0.000) | (0.015) | (0.000) | (0.018) |
| Economic Losses | . | 0.897*** | . | 1.138*** |
| | | (0.314) | | (0.271) |
| Economic losses × Infected (last 14 days) | . | 0.002 | . | −0.004 |
| | . | (0.031) | . | (0.036) |
| Individual controls | Yes | Yes | Yes | Yes |
| Observations | 2768 | 2768 | 2154 | 2154 |
| Individuals | 1025 | 1025 | 718 | 718 |
| R2 | 0.977 | 0.015 | 0.983 | 0.016 |

The table shows the results of regression with individual fixed effects. In the first and third columns, the dependent variable measures economic losses due to COVID-19. In the second and fourth columns, the dependent variable measures preferences for redistribution. The variable of interest is the logarithm of the average number of COVID-19-related cases per 100,000 inhabitants at the county level in the 14 days prior to the interview. Individual controls are gender, age and education groups, partnership/marital status, parental status, health, employment status, and Covid perception

Standard errors in parentheses *** $p < 0.01$, ** $p < 0.05$, * $p < 0.1$

pandemic on redistributive preferences, we present some additional evidence from our survey in the following subsection.

Because being able to control for individual fixed effects is a great advantage of the survey we use, we only show and comment on results from both balanced and unbalanced panels in the remainder of the paper. Indeed, while the former allows us to track the effect over all periods, the latter has a larger sample size. However, the results are qualitatively similar in most of the models, showing that attrition bias is not a relevant issue in the present analysis.

### 5.1 Possible mechanisms

**Economic losses** If the severity of the pandemic directly negatively affects individuals' incomes, we can expect this to be reflected in a shift in support for redistribution. The survey includes a question asking whether the respondent suffered economic losses caused by the pandemic crisis. This question is asked only to employed workers, so our sample is almost halved; however, it can help us to understand the relationship between preferences for redistribution and COVID-19-related economic losses. Our results are presented in Table 3. As expected, the more severe the pandemic, the more likely individuals are to also have directly experienced an income loss. In turn, on average, individuals who experienced a direct income shock are more in favor of redistribution, but they do not react differently to the collective shock brought by the pandemic.

**Inequality aversion** A health crisis like the one we have been experiencing due to the COVID-19 pandemic may have affected people's normative views about inequality,

**Table 4** Inequality aversion

| | (1) | (2) | (3) | (4) | (5) | (6) |
|---|---|---|---|---|---|---|
| | FE unbalanced | | | FE balanced | | |
| | Pie rich | Pie poor | All same condition | Pie rich | Pie poor | All same condition |
| Incidence rate (last 14 days) | −0.275*** | 0.102*** | 0.023** | −0.273*** | 0.102*** | 0.026** |
| | (0.013) | (0.014) | (0.011) | (0.015) | (0.015) | (0.012) |
| Individual controls | Yes | Yes | Yes | Yes | Yes | Yes |
| Observations | 6176 | 6176 | 4058 | 4932 | 4932 | 3258 |
| Individuals | 2266 | 2266 | 2029 | 1644 | 1644 | 1629 |
| R2 | 0.111 | 0.018 | 0.013 | 0.108 | 0.017 | 0.019 |

The table shows the results of regression with individual fixed effects. The dependent variable in the first and fourth (second and fifth) columns is built around a question asking how much should be allocated to the people in the top (bottom) 20% of earnings. The dependent variable in the third and sixth columns is built around a question asking whether a society where everyone has the same condition can be considered fair. The variable of interest is the logarithm of the average number of COVID-19-related cases per 100,000 inhabitants at the county level in the 14 days prior to the interview. Individual controls are gender, age and education groups, partnership/marital status, parental status, health, employment status, and Covid perception

Standard errors in parentheses *** $p < 0.01$, ** $p < 0.05$, * $p < 0.1$

changing, for example, individuals' beliefs over the reasons behind the income gaps between the rich and the poor. If the pandemic has shifted the share of inequality that people believe comes from bad luck instead of a lack of effort, we should see a shift also in inequality aversion.

We investigate this channel by regressing our measure of inequality aversion, introduced at the end of Section 3, on COVID-19 cases. Results are presented in Table 4. In line with the theoretical argument introduced above, we find that an increase in COVID-19 cases leads people to think that less wealth should be allocated to the top 20% and more wealth to the bottom 20%, showing an increase in their inequality aversion. Consistent with these results, we also find that an increase in COVID-19 infection rates pushes people to believe that a society is fair if everyone has the same living conditions.[8]

**Perception of inequality of opportunity** Another possible channel through which the pandemic can affect preference for redistribution is by changing not only the normative view on inequality but also the perception of it. In our survey, we only have a measure of perception of income inequality in the first wave, but we have questions in all waves gathering people's perception of equality of current and intergenerational opportunities. As shown in Table 5, we find that an increase in COVID-19 infection rates increases individuals' perception of inequality of opportunities. An increase in the COVID-19 infection rates also increases support for the belief that future generations will have fewer opportunities

[8] These results are different from other studies such as Asaria et al. (2021) and Cappelen et al. (2021). However, these studies differ in many aspects, such as the definition of the treatment variable, the cultural context, and the period of investigation, so the differences are not unexpected.

**Table 5** Perceptions of inequality of opportunity

| | (1) FE unbalanced | (2) | (3) FE balanced | (4) |
|---|---|---|---|---|
| | Everyone has the same opp. | Future gen. Same opp. | Everyone has the same opp. | Future gen. Same opp. |
| Incidence rate (last 14 days) | −0.021** (0.008) | −0.016* (0.009) | −0.029*** (0.011) | −0.024** (0.011) |
| Individual controls | Yes | Yes | Yes | Yes |
| Observations | 6794 | 6652 | 4209 | 4209 |
| Individuals | 2625 | 2616 | 1403 | 1403 |
| R2 | 0.007 | 0.007 | 0.009 | 0.009 |

The table shows the results of regression with individual fixed effects. The dependent variable in the first (third) column measures the perception that in Germany, everyone has the same opportunities. The dependent variable in the second (fourth) column measures the perception that in Germany, future generations will have the same opportunities as current generations. The variable of interest is the logarithm of the average number of COVID-19-related cases per 100,000 inhabitants at the county level in the 14 days prior to the interview. Individual controls are gender, age and education groups, partnership/marital status, parental status, health, employment status, and Covid perception

Standard errors in parentheses *** $p < 0.01$, ** $p < 0.05$, * $p < 0.1$

in life. Thus, the worsening of the pandemic increases both the taste for equality and the perception of it.

These results seem at odds with our results on preferences for redistribution and might suggest that inequality aversion and perception of equal opportunities do not necessarily go hand in hand with preferences for redistribution.

**Interpersonal trust** Research shows that the COVID-19 pandemic might decrease interpersonal trust (Brück et al. 2020). Because the literature has established a nexus between generalized trust and support for the welfare state (Algan et al. 2016; Daniele and Geys 2015), it may be that part of the negative effect between the intensity of the pandemic and preferences for redistribution is due to a decrease in interpersonal trust. We investigate this possible channel by regressing our measure of interpersonal trust on the intensity of the COVID-19 pandemic. Results in Table 6 show that an increase in the intensity of the pandemic has no significant effects on interpersonal trust in our sample. This evidence appears to rule out the possibility that our main results are driven by a decrease in interpersonal trust.

**Trust in political institutions** A possible explanation for our puzzling results might be that the pandemic, while increasing both the need for compensation and the aversion to inequality, has decreased the level of trust in the same institutions that are in charge of redistributive policies. The literature has shown that lack of trust in institutions might decrease the support for redistribution (Algan et al. 2011; Daniele et al. 2020). Kuziemko et al. (2015) show that views about inequality do not necessarily have an impact on policy preferences, partly because of people's low level of institutional trust. To show whether this is a plausible explanation in our context, we look at the impact of the intensity of the pandemic on trust in the different political institutions described in Section 3. The results displayed in

**Table 6** Interpersonal trust

| | (1) Social trust (unbalanced) | (2) Social trust (balanced) |
|---|---|---|
| Incidence rate (last 14 days) | 0.018 | 0.020 |
| | (0.012) | (0.013) |
| Individual controls | Yes | Yes |
| Observations | 6176 | 4932 |
| Individuals | 2266 | 1644 |
| R2 | 0.011 | 0.013 |

The table shows the results of regression with individual fixed effects. The dependent variable measures interpersonal trust. The variable of interest is the logarithm of the average number of COVID-19-related cases per 100,000 inhabitants at the county level in the 14 days prior to the interview. Individual controls are gender, age and education groups, partnership/marital status, parental status, health, employment status, and Covid perception

Standard errors in parentheses *** $p < 0.01$, ** $p < 0.05$, * $p < 0.1$

Table 7 show that COVID-19 has an effect on institutional trust. The worsening of the pandemic decreased trust in the federal government by about 13 percentage points, which represents about 3.2% of the mean. Furthermore, we find evidence that an increase in COVID-19 cases also decreases trust in Parliament and in state governments by a similar amount.

These results suggest that the decrease in support for redistribution is not driven by an increase in the acceptance of income difference, as, if anything, we have shown an increase in inequality aversion, nor by a lack of reaction to direct income shocks, but could be partly due to a general decrease in trust towards political institutions.

To further investigate this aspect, we split our sample between individuals declaring a high level or a low level of trust. For completeness, we show the results both concerning high (low) levels of institutional trust and interpersonal trust.[9] The results displayed in Table 8 show very interesting differences driven by the level of trust. Specifically, we find that the negative effect of COVID-19 on redistribution is highly significant only among individuals who have on average a low level of institutional and interpersonal trust. This is also true when we look at the interaction model instead of splitting the sample, as shown in Fig. 3. These findings are particularly interesting because of their consistency with the literature and because they provide a convincing reason why the rise of inequality aversion due to the pandemics is not coupled with a similar increase in propensity for redistribution.

[9] Individuals with a high (low) level of trust are defined as individuals scoring on average more (less) than 4 in our measure related to individual trust and scoring on average more (less) than 5 in our measure of interpersonal trust.

**Table 7** Institutional trust

| | (1)<br>Fed. gov. | (2)<br>FE unbalanced Parliament | (3)<br>State gov. | (4)<br>Fed. gov. | (5)<br>FE balanced Parliament | (6)<br>State gov. |
|---|---|---|---|---|---|---|
| Incidence rate (last 14 days) | −0.124*** | −0.094*** | −0.095*** | −0.136*** | −0.104*** | −0.108*** |
| | (0.007) | (0.007) | (0.007) | (0.007) | (0.008) | (0.008) |
| Individual controls | Yes | Yes | Yes | Yes | Yes | Yes |
| Observations | 6176 | 6176 | 6176 | 4932 | 4932 | 4932 |
| Individuals | 2266 | 2266 | 2266 | 1644 | 1644 | 1644 |
| R2 | 0.083 | 0.055 | 0.051 | 0.094 | 0.063 | 0.064 |

The table shows the results of regression with individual fixed effects. The dependent variables measure trust in political institutions (Federal government in col.(1) and (4), Parliament in col.(2) and (5), State government in col.(3) and (6)). The variable of interest is the logarithm of the average number of COVID-19-related cases per 100,000 inhabitants at the county level in the 14 days prior to the interview. In- dividual controls are gender, age and education groups, partnership/marital status, parental status, health, employment status, and Covid perception

Standard errors in parentheses *** $p < 0.01$, ** $p < 0.05$, * $p < 0.1$

**Table 8** Sample split according to trust

| | (1)<br>Low inst. Trust | (2)<br>High inst. Trust | (3)<br>Low social trust | (4)<br>High social trust |
|---|---|---|---|---|
| Incidence rate (last 14 days) | −0.037** | −0.010 | −0.030** | −0.009 |
| | (0.018) | (0.014) | (0.013) | (0.018) |
| Individual controls | Yes | Yes | Yes | Yes |
| Observations | 1896 | 2610 | 2958 | 1623 |
| Individuals | 632 | 870 | 986 | 541 |
| R2 | 0.028 | 0.014 | 0.014 | 0.020 |

The table shows the results of regression with fixed effects. The dependent variable measures preferences for redistribution. The results are shown for different samples depending on the level of trust in state government (first two columns) and trust in other people (last two columns). The sample is split at the central level of the Likert scales. Individual controls are gender, age and education groups, partnership/marital status, parental status, health, employment status, and Covid perception

Standard errors in parentheses *** $p < 0.01$, ** $p < 0.05$, * $p < 0.1$

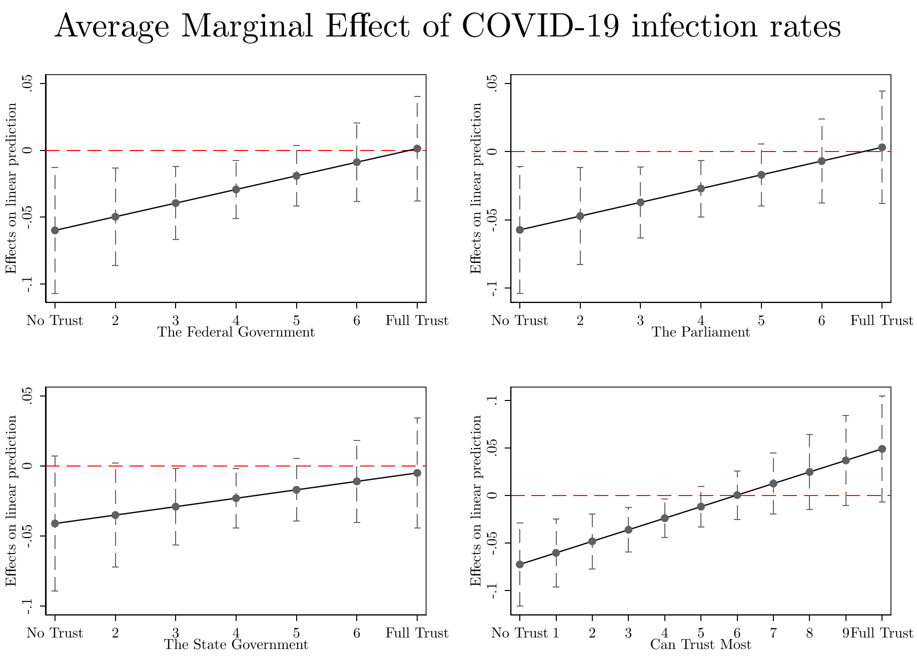

**Fig. 3** Redistribution (main variable) . Average Marginal Effect of COVID-19 infection rates. Notes: The figure reports the coefficients of the incidence rate at each level of trust. The confidence interval is at the 5% level

## 5.2 Robustness checks

**Alternative redistribution variable** Our survey contains a second variable that could be used to measure individuals' preference for redistribution. Different from the main outcome of the paper, the wording of this second variable does not put specific emphasis on the role of the government. As displayed in the first column of Table 9, on average we find no effect of an increase in severity of the crisis on this second measure of redistribution. However, similarly to the result shown above, there is an interesting heterogeneity across individual trust. Columns 2 to 5 of Table 9 and Fig. 4 show that on the one side, there is a significant negative effect for the individuals with lower trust in the institutions, while on the other side, we see a positive effect for individuals with high trust, suggesting that the role of trust is even more pronounced in this case. Overall, people's trust in institutions and in other people has a consistent and significant effect in explaining the link between the shock and the preferences for redistribution: the less individuals trust the government and other people, the more the shock decreases their redistribution preferences and vice-versa.

**Alternative measures of severity of COVID-19** To ensure that our results are not sensitive to the choice of the measure of COVID-19 severity used, we test for alternative measures. First, we use the number of deaths in the last 14 days. Second, because the choice of analyzing 14 days before the date of the interview is arbitrary, we replicate the analysis using a time span of 0, 7, 28, and 90 days. Table 10 shows that results are unchanged if we use these alternative measure and time spans. This may reinforce the assumption made in Section 4 that the measure we use captures the perceived severity of COVID-19 spread in society, and the results do not depend on the specific measure.

**Alternative error clustering levels** Finally, a possible threat to our identification is that other time-varying factors correlated to the development of the COVID-19 pandemic at the county or state level are driving our results. To rule out this possibility, we cluster the standard errors at the county and state level (Table 11) and observe that the results are substantially unchanged. Because some individuals move to a different county over time, we cannot cluster at the county level when we introduce the individual fixed effects, as the clusters are not nested within the panel. Therefore, we include the county-fixed effects in our regressions, and we cluster the standard errors at the state level to control for those who

**Table 9** Preference for redistribution (alternative variable)

| | (1)<br>All | (2)<br>Low inst. Trust | (3)<br>High inst. Trust | (4)<br>Low social trust | (5)<br>High social trust |
|---|---|---|---|---|---|
| Incidence rate (last 14 days) | 0.005 | −0.046* | 0.026 | −0.029 | 0.063** |
| | (0.016) | (0.028) | (0.020) | (0.022) | (0.025) |
| Individual controls | Yes | Yes | Yes | Yes | Yes |
| Observations | 4932 | 1896 | 2610 | 2958 | 1623 |
| Individuals | 1644 | 632 | 870 | 986 | 541 |
| R2 | 0.004 | 0.018 | 0.014 | 0.008 | 0.014 |

The table shows the results of linear regressions with individual fixed effects. The dependent variable measures preferences for redistribution using a different wording with respect to our main variable. The results are shown for different samples depending on the level of trust in state government (col.(2)–(3)) and trust in other people (col.(4)–(5)). The sample is split at the central level of the Likert scales. The variable of interest is the logarithm of the average number of COVID-19-related cases per 100,000 inhabitants at the county level in the 14 days prior to the interview. Individual controls are gender, age and education groups, partnership/marital status, parental status, health, employment status, and Covid perception

Standard errors in parentheses *** $p < 0.01$, ** $p < 0.05$, * $p < 0.1$

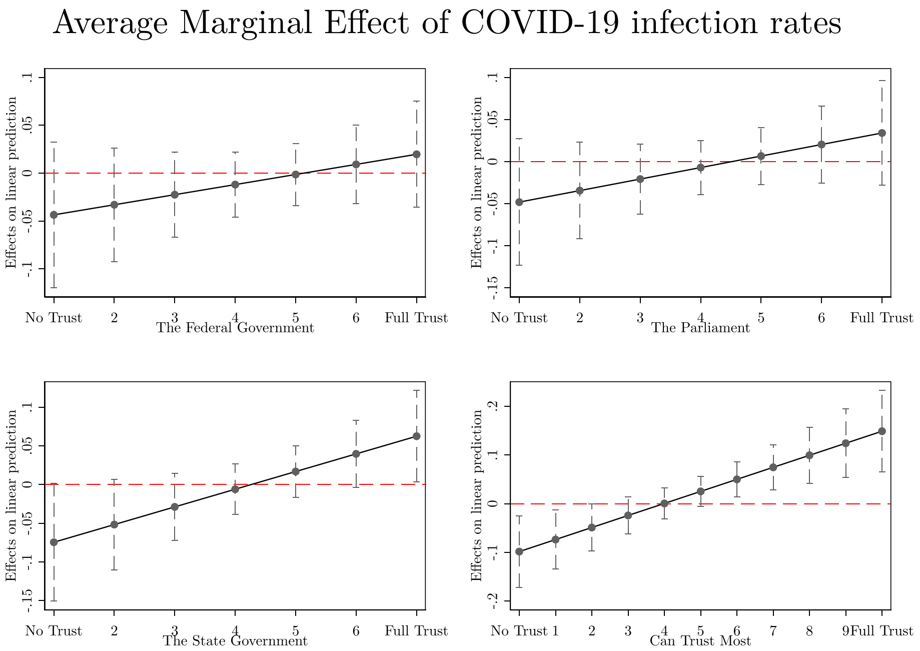

**Fig. 4** Preference for redistribution (alternative variable) . Average Marginal Effect of COVID-19 infection rates. Notes: The figure reports the coefficients of the incidence rate at each level of trust. The confidence interval is at the 5% level

**Table 10** Robustness checks

| | (1) | (2) | (3) | (4) | (5) |
|---|---|---|---|---|---|
| Death rate (last 14 days) | −0.069* | . | . | . | . |
| | (0.036) | . | . | . | . |
| Incidence rate (daily) | . | −0.025* | . | . | . |
| | . | (0.013) | . | . | . |
| Incidence rate (last 7 days) | . | . | −0.021** | . | . |
| | . | . | (0.010) | . | . |
| Incidence rate (last 28 days) | . | . | . | −0.026** | . |
| | . | . | . | (0.012) | . |
| Incidence rate (last 90 days) | . | . | . | . | −0.038* |
| | . | . | . | . | (0.020) |
| Individual controls | Yes | Yes | Yes | Yes | Yes |
| Observations | 4932 | 4932 | 4932 | 4932 | 4932 |
| Individuals | 1644 | 1644 | 1644 | 1644 | 1644 |
| R2 | 0.008 | 0.008 | 0.008 | 0.008 | 0.008 |

The table shows the results of regression with fixed effects. The dependent variable measures preferences for redistribution. The variable of interest is the logarithm of the average number of COVID-19-related deaths per 100,000 inhabitants or the number of COVID-19 cases at the county level at different day intervals prior to the interview. Individual controls are gender, age and education groups, partnership/marital status, parental status, health, employment status, and Covid perception

Standard errors in parentheses *** $p < 0.01$, ** $p < 0.05$, * $p < 0.1$

**Table 11** Preference for redistribution (clustered standard errors)

| | (1)<br>OLS | (2)<br>OLS | (3)<br>FE unbalanced | (4)<br>FE balanced |
|---|---|---|---|---|
| Incidence rate (last 14 days) | −0.031***<br>(0.009) | −0.025***<br>(0.009) | −0.021*<br>(0.011) | −0.020*<br>(0.011) |
| Individual controls | No | Yes | Yes | Yes |
| Landkreis FE | No | No | No | Yes |
| Observations | 6176 | 6167 | 4842 | 4842 |
| Individuals | | | 1614 | 1614 |
| R2 | 0.001 | 0.057 | 0.007 | 0.013 |

The table shows the results of regression with and without fixed effects. Standard errors are clustered at the county level in col.(1) and (2) and at the state level in col.(3) and (4). The sample size is smaller in col.(1) and (2) because individuals may change their county of residence. In this case, it is not possible to include these individuals in the regression with standard errors clustered at the county level. Individual controls are gender, age and education groups, partnership/marital status, parental status, health, employment status, and Covid perception

Standard errors in parentheses *** $p < 0.01$, ** $p < 0.05$, * $p < 0.1$

move to a different county and eliminate possible sorting behavior at both the county and the state level.

## 6 Conclusions

Our work sheds light on how collective negative shocks affect preferences for redistribution. Specifically, we draw on the county-level heterogeneous spread of the COVID-19 pandemic in Germany to understand how an increase in its intensity influences people's preferences for redistribution. The empirical strategy allows us to control for both time-variant and time-invariant individual characteristics and uncover a causal relationship between exogenous adverse shocks and preferences for redistribution.

We show that an increase in the intensity of the pandemic leads to a decrease in the support for income redistribution. We test different channels that might explain our findings. We investigate whether the COVID-19 crisis affects people's normative views on inequality, and we find that an increase in the intensity of the pandemic increases inequality aversion. Consistent with standard economic theory, we also find that those who incurred economic losses are more likely to support redistribution, while we do not find any effect of the pandemic on interpersonal trust.

A possible explanation of our results is that people's lower support for redistribution strongly depends on the individual's level of trust. We show indeed that an increase in the intensity of the current pandemic decreases the level of trust in the state government, the federal government, and Parliament. We dig deeper into this possible mechanism by interacting the incidence rate with the level of trust and by splitting the sample between individuals with a high or low level of trust. We find that the effect of the COVID-19 pandemic on preferences for redistribution is mainly driven by people with a low level of trust. The lack of trust towards the institutions in charge of the redistributive policies might represent a reason why people are at the same time less supportive of redistribution and more averse to inequality (Kuziemko et al. 2015).

Previous research has investigated how adverse shocks such as earthquakes or the COVID-19 crisis might affect peoples' support for redistribution; however, the drivers behind this relationship are unclear (Cappelen et al. 2021; Daniele et al. 2020; Gualtieri et al. 2019). Our work builds on past research so to provide a comprehensive picture of the mechanisms that might affect the relationship between negative shocks and preferences for redistribution. Our findings seem to suggest that, when confronted with such unexpected events, people's aversion to inequality and redistributive preferences do not necessarily go hand in hand.

## Appendix: The survey

**Funding** Open access funding provided by Università degli Studi di Pavia within the CRUI-CARE Agreement. This project is funded by the Deutsche Forschungsgemeinschaft (DFG - German Research Foundation) under Germany's Excellence Strategy-EXC-2035/1 – 390681379, as well as a financial contribution from the Italian Ministry of University and Research PRIN 2017K8ANN4 "New approaches to political economy: from methods to data" is gratefully acknowledged.

## Declarations

**Ethics** The paper raises no ethical issues. The survey received an ethical waiver from the Ethics Committee (Institutional Review Board, IRB) of the University of Konstanz, RefNo: IRB20KN09-006.

**Conflict of interest** The author declares that he has no conflict of interest.

**Open Access** This article is licensed under a Creative Commons Attribution 4.0 International License, which permits use, sharing, adaptation, distribution and reproduction in any medium or format, as long as you give appropriate credit to the original author(s) and the source, provide a link to the Creative Commons licence, and indicate if changes were made. The images or other third party material in this article are included in the article's Creative Commons licence, unless indicated otherwise in a credit line to the material. If material is not included in the article's Creative Commons licence and your intended use is not permitted by statutory regulation or exceeds the permitted use, you will need to obtain permission directly from the copyright holder. To view a copy of this licence, visit http://creativecommons.org/licenses/by/4.0/.

## Data Availability

The data that support the findings of this study are (will be) accessible via the data archive of GESIS—The Leibniz Institute for the Social Sciences at https://data.gesis.org/sharing/#!Search/?partner=exzclu.

## Appendix: The survey

To gain a better understanding of how people in Germany cope with the social and political effects of the COVID-19 crisis, a group of researchers at the “The Politics of Inequality" cluster of excellence at the University of Konstanz initiated a multifaceted survey program. One of the authors of this paper is a member of this group. The issues covered in this program are diverse. A total of 18 Cluster researchers from various disciplines are involved in this project, which brings together the Cluster’s research areas and disciplines. The program is coordinated by the Methods Hub, a central institution that provides Cluster researchers with method skills and practical support. The data are collected by the University of Konstanz survey-LAB.

The online surveys were implemented in three waves. A first wave was collected during April- June 2020, with over 8,000 participants. The second wave of 7,000 interviews was collected in October-November 2020 and features a panel setting. In May 2021, we conducted a third survey wave with more than 6,000 participants. The Kantar panel (a permanent group of respondents) was used for topics that required a particularly representative sample of the population, like the one in this paper. The Respondi panel was used for other research questions that rely on a large number of cases. Based on the Open Data strategy, the data collected as part of this survey program are free to use for scientific purposes after a short embargo. They are (will be) accessible via the data archive of GESIS—The Leibniz Institute for the Social Sciences at https://data.gesis.org/sharing/#!Search/?partner=exzclu.

Comprehensive information on the survey program, descriptions of the topic-oriented modules and their results, and complete information on methods and the underlying data are available on https://www.exc.uni-konstanz.de/en/inequality/research/covid-19-and-inequality-surveys-program/. An English translation of the three questionnaires for the Kantar panel used in this paper is available at https://www.exc.uni-konstanz.de/en/inequality/research/covid-19-and-inequality-surveys-program/documentation/.
